# Adaptive Weighted Strategy Based Integrated Surrogate Models for Multiobjective Evolutionary Algorithm

**DOI:** 10.1155/2022/5227975

**Published:** 2022-06-25

**Authors:** Ke Bao, Wei Fang, Yourong Ding

**Affiliations:** ^1^Wuxi Institute of Technology, Wuxi, Jiangsu 214121, China; ^2^Jiangnan University, Wuxi, Jiangsu 214122, China

## Abstract

Although the integrated model has good convergence ability, it is difficult to solve the multimodal problem and noisy problem due to the lack of uncertainty evaluation. Radial basis function model performs best for different degrees of nonlinear problems with small-scale and noisy training datasets but is insensitive to the increase of decision-space dimension, while Gaussian process regression model can provide prediction fitness and uncertainty evaluation. Therefore, an adaptive weighted strategy based integrated surrogate models is proposed to solve noisy multiobjective evolutionary problems. Based on the indicator-based multiobjective evolutionary framework, our proposed algorithm introduces the weighted combination of radial basis function and Gaussian process regression, and U-learning sampling scheme is adopted to improve the performance of population in convergence and diversity and judge the improvement of convergence and diversity. Finally, the effectiveness of the proposed algorithm is verified by 12 benchmark test problems, which are applied to the hybrid optimization problem on the construction of samples and the determination of parameters. The experimental results show that our proposed method is feasible and effective.

## 1. Introduction

With the continuous upgrading of application requirements, many problems in our real-world have been abstracted into high-dimensional multiobjective optimization problems [1]. For example, regarding the Internet of Vehicles, problems such as intrusion detection faced by the in-vehicle control network, software product selection in software engineering, selection and distribution of relief supplies, and green production optimization in coal industry can be modeled as high-dimensional objective optimization problems [2]. These practical problems may involve one or more conflicting optimization objectives, which are frequently constrained by harsh constraints. The selection of optimization schemes and the performance of optimization algorithms have become constant challenges as the complexity of the problem has increased [3].

Therefore, in recent years, how to use multiobjective evolutionary algorithm to solve high-dimensional objective optimization problems has attracted more and more attention. The existing multiobjective evolutionary algorithms are generally divided into the following three categories: Pareto domination-based [4,5], indicator-based [6], and decomposition-based [7] multiobjective evolutionary algorithm. The diversity maintenance mechanism of most Pareto domination-based multiobjective evolutionary algorithms is only applicable to Pareto-front optimization problems with regular distribution [8]. When the distribution of Pareto-front is irregular or complex, those algorithms are often difficult to obtain solutions that consider proximity, distribution, and malleability. For index-based multiobjective evolutionary algorithms, they usually choose the optimal solution that makes more contributions to the index, which makes the dominated solution more likely to be favored than the nondominated solution; decomposition-based multiobjective evolutionary algorithms often have difficulty determining the orientation vectors that are matching with the shape of irregular Pareto-front, which leads to hardly solve the complex Pareto-front multiobjective optimization problems. Thus, to solve the previously mentioned problems, a variety of effective high-dimensional multiobjective evolutionary algorithms (MaOEAs) have emerged [9]. Some improved MaOEAs choose more loose Pareto dominance criteria or simultaneously adopt Pareto dominance criteria and convergence criteria to alleviate the selection pressure [10]. Although this kind of algorithms can better ensure the convergence of the obtained Pareto optimal solution set, it is likely to ingrain a Pareto-front with poor distribution. On the other hand, mops problems are often mixed with noise in the process of fitness evaluation, which may mislead the search direction and reduce the optimization efficiency [11]. From this point of view, noise interferes with the steps of multiobjective optimization, such as determining nondominated individuals, diversity preservation, and elitism, so that the optimal solution of multiobjective optimization problems cannot be obtained, and the optimization problems cannot be well solved [11]. Therefore, how to deal with the noise in multiobjective optimization problem becomes very critical. However, noisy multiobjective optimization problems are general in practical engineering fields.

It can be seen from the previously mentioned analysis that most of the existing classical multiobjective evolutionary algorithms show bound limitations in dealing with high-dimensional objective optimization problems [12]. Therefore, many scholars combine multiobjective optimization with denoising model to solve the noise in the multiobjective optimization problems, resulting in noise multiobjective optimization algorithm. Because the existence of noise affects the inaccuracy of individual selection, adjusting the selection process is a fateful method to deal with the noise multiobjective optimization problem [13]. General selection methods include nondominated sorting selection, probabilistic sorting selection, threshold selection, and so on. Optimization problems in real-world are often affected by noise. And with different distributions, constraints, and central trends, noise may appear in random model parameters, objective functions, and decision variables. Many literatures have recently adopted evolutionary computation method to solve the optimization problems in practical applications from different perspectives [14]. However, for the noise optimization problems existing in engineering problems, even if the variables are set to constant, different target values will be obtained from multiple target evaluation processes due to the noise doping in the evaluation process of fitness function. In this case, the individuals entering the next generation are often not high-quality individuals [15]. To solve these problems, many researchers have proposed a variety of noise optimization algorithms.

Intuitively, a common method to reduce noise interference is to average multiple object values obtained from multiple function evaluation processes and then use the final average value to approximate the real object value. Eskandari and Geiger [16] used a fixed number of samples and averaged the target values of them and then provided a new sufficient condition for the convergence of the evolutionary algorithm that is with fixed number of samples. If there are many samples, there will be a colossal computational cost in function evaluation. Basseur and Zitzler [17] proposed a strategy to reduce the evaluation times for everyone and not the average for all individuals; they averaged the time of some of the best individuals. So, a better solution can be found by averaging a small number of function evaluations. This simple method can significantly reduce the number of function evaluations. However, self-adaptive sampling of data can reduce the influence of noise, but sometimes it may have a great computational cost.

Therefore, some scholars have adopted one threshold method in the deterministic selection of evolutionary strategies. If and only if the fitness value of a descendant is better than that of its parent, the descendant will be accepted [18]. This kind of method calculates the probability that an individual dominates all individuals, the probability that an individual is dominated by all individuals, and the probability that it has no dominance relationship with all individuals, to calculate the rank value of this individual. This mechanism greatly improves the correctness of individual ranking and proves that the hierarchical ranking scheme used by NSGA in the case of no-noise may have defects in dealing with noisy problems [19]. Shim et al. proposed a regularity model-based noise multiobjective optimization algorithm according to regularity model-based multiobjective estimation of distribution algorithm [20], where the nondominated solution is used to establish the Pareto-solution model and samples the sample solution from it. With the continuous optimization of the evolution process, the established model continues to approach the real Pareto-solution, and the quality of the sample solution on the model is gradually improved, which proves that the model has a certain ability to resist noise. Hong et al. proposed the strategy that dividing the search space into several nonoverlapping hyper-spheres and moving individual solutions in each sphere, which improved the average performance of the spheres [21]. This local model can filter noise and increase the robustness of the algorithm. By combining cooperative evolutionary frame and differential evolutionary algorithm, namely, cooperative differential coevolution algorithm, this model can effectively solve large-scale multiobjective optimization problems [22]. Multilevel cooperative coevolution algorithm adopts a technology called random grouping to group interactive variables into a subcomponent. In addition, another technique called self-adaptive weighting is used to adaptively adjust the subcomponents. In the cooperative coevolution with variable interaction learning [23], a new coevolution framework is proposed. Initially, all variables are regarded as independent variables, and each variable is placed in a separate group. And in the iterative process, the relationship between variables is gradually found and merged accordingly. DG2 is an improved differential grouping algorithm, which has better efficiency and grouping accuracy [24]. In the algorithm of using variable analysis method to reduce the dimension of search space, multiobjective evolutionary algorithm based on decision variable analysis controls variable analysis to identify the conflict between objective functions [25]. The algorithm carries out which variables affect the diversity of the generated solutions and which variables play an important role in the overall convergence. Through the analysis of interdependent variables, the original multiobjective optimization problems are transformed into a series of sublevel multiobjective optimization problems, which can improve the convergence of most difficult multiobjective optimization problems. In order to reduce the dimension of search space through problem transformation, the weighted optimization framework is one of the most representative algorithms, where decision variables are divided into multiple groups, and each group is assigned a weight vector [21–24]. Thus, the optimization of original decision variables can be transformed into the optimization of weight vector. After the problem is transformed, the decision variable space of new problems will be greatly reduced. Large-scale multiobjective optimization framework also adopts the problem transforming strategy, which firstly decompose Pareto-solution into two directions associated with the weight vector, and then the weight vector is taken as input to construct a series of subproblems and is designed to track the corresponding points on the Pareto-solution so as to reduce the dimension of decision variable space [18,21,22,24].

Although the integrated model has good convergence ability, it is difficult to solve the multimodal problem and noisy problem due to the lack of uncertainty evaluation. Therefore, an adaptive weighted strategy based integrated surrogate model is proposed to solve noisy multiobjective evolutionary problems in this paper. Based on the indicator-based multiobjective evolutionary framework, our proposed algorithm introduces the weighted combination of radial basis function and Gaussian process regression, and U-learning sampling scheme is adopted to improve the performance of population in convergence and diversity and judge the improvement of convergence and diversity. Finally, the effectiveness of the proposed algorithm is verified by 12 benchmark test problems, which are applied to the hybrid optimization problem on the construction of samples and the determination of parameters. The experimental results show that our proposed method is feasible and effective.

## 2. Multiobjective Optimization and Its Noisy Problem

For a *n* multidimensional decision variable, the mathematical model of multiobjective optimization problem with *m*dimension can be defined as follows:

(1) $$\begin{array}{c} \text{min}F\left(x\right)={\left(f_{1}\left(x\right),f_{2}\left(x\right),\cdots ,f_{m}\left(x\right)\right)}^{T}s.tG_{i}\left(x\right)\geq 0,\\ i\in 1,2,\ldots ,pH_{j}\left(x\right)=0,j\in 1,2,\ldots ,q, \end{array}$$

where *x*=(*x*_1_, *x*_2_,…, *x*_*n*_) ∈ Ω; *x* is the decision variable and Ω is the decision-space Ω=∏_*i*=1_^*n*^[*L*_*i*_, *U*_*i*_]; *L*_*i*_ and *U*_*i*_ are the upper and lower boundaries of the *x*_*i*_; *F*(*x*) is the object vector, representing the mapping relationship of Ω⟶*R*^*m*^; *G*(*x*) and *H*(*x*) are the constraints of the problem. Given *x*^1^=(*x*_1_^1^, *x*_2_^1^,…, *x*_*n*_^1^) and *x*^2^=(*x*_1_^2^, *x*_2_^2^,…, *x*_*n*_^2^), they are two decision vectors satisfying constraints in the object space, and *x*^1^ Pareto dominates *x*^2^, denoted as *x*^1^≻*x*^2^, which satisfied (∀*i*)*f*_*i*_(*x*^1^) < =*f*_*i*_(*x*^2^), *i* ∈ {1,…, *m*}.

With the increasing demand of engineering problems, the results of solving multiobjective optimization problems are not only satisfied with a Pareto solution set or a Pareto optimal solution based on the preference of decision-makers. Sometimes, due to the influence of surrounding environmental factors or noise interference, people prefer to get a more robust Pareto optimal solution set or Pareto optimal solution. The so-called robustness is simply the sensitivity of the objective function to the small disturbance in the decision parameters [25]. If a global optimal solution is very sensitive to the variable disturbance, the final optimal solution obtained in reality may correspond to a different optimal value from the theoretical solution, which means a Pareto solution set with poor robustness is generated. To facilitate analysis and description, we will introduce the noisy model for the multiobjective optimization problem and proposed a simple and novel integrated surrogate-assisted model.

Since the evolutionary algorithm is less affected by noise in the early stage, Gaussian regression value can be used as the denoising object value. In practical engineering applications, the observed response usually contains noise, namely, *y*(*x*)=*f*(*x*)+*n*. Generally, the noise is assumed to be zero mean Gaussian distribution *ε* ~ *N*(0, Σ_*n*_), where the common form of Σ_*n*_ is Σ_*n*_=*σ*_*n*_**I**, and **I** is the identity matrix. For the responses of all observed samples, the variance of their noise is the same. That means the noise is an independent and identically distributed Gaussian distribution.

To reduce the influence of noise, a noise estimation and denoising method is proposed. The joint Gaussian distribution of the prediction *y*(*x*) and noisy response Γ at *x* is written as follows:

(2) $$\begin{array}{c} \left\{\begin{array}{c} \Gamma \\ y\left(x\right) \end{array}\right\}∼N\left(\left\{\begin{array}{c} u\\ u\left(x\right) \end{array}\right\},\left\{\begin{array}{c} \sigma^{2}R+\Sigma_{n}\sigma^{2}r\left(x\right)\\ \sigma^{2}r^{T}\left(x\right)+I \end{array}\right\}\right), \end{array}$$

where the variance matrix is C=*σ*^2^*R*+Σ_*n*_ and the covariance vector is *c*=*σ*^2^*r*^*T*^(*x*).

## 3. Surrogate-Assisted Evolutionary Algorithm

**Figure 1:**
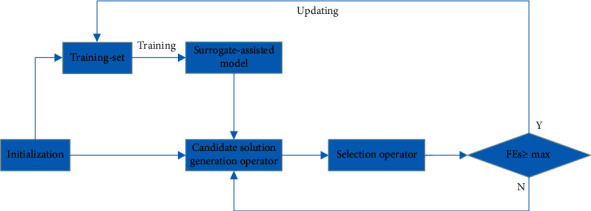
The framework of surrogate-assisted evolutionary algorithm.

**Figure 2:**
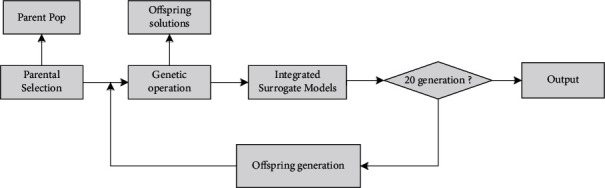
The framework of offspring generation.

Surrogate-assisted model is to construct a relatively simple function from a complex function by collecting feature points and optimize the new function to obtain the optimal solution of complex function, which is shown in Figure 1. Since the surrogate-assisted model can only represent the real model to a certain extent, so its optimal solution can not directly represent the optimal solution of the original objective function. It is necessary to update the surrogate-assisted model according to a certain criterion. The framework of offspring generation is shown in Figure 2.

The search strategy of surrogate-assisted evolutionary algorithm (SAEAs) in the optimization process largely depends on a surrogate model [21,26]. The reason is that the surrogate model assumes that it can provide sufficiently accurate function estimation. Since the surrogate model cannot provide the same properties as the original objective function, it may even produce the optimal value that does not actually exist. Therefore, how to select an appropriate surrogate model is a very important step in surrogate-assisted evolutionary algorithm. In practical application, the conventional operation is to use the surrogate model together with object-function evaluation. This section will briefly analyze two main surrogate-assisted models: Gaussian process regression(GPR) [27–30] and radial basis function (RBF) [25, 31].

### 3.1. Gaussian Process Regression

Gaussian process regression is a Bayesian statistical method for modeling functions, which is very suitable for objective function evaluation. Gaussian process does not need predefined data-structure and can approximate the nonlinear, discontinuous, and multimodal functions, which provides an uncertainty measurement in the form of standard deviation for the prediction function [32]. Gaussian process is the extension of multivariate Gaussian distribution to infinite dimensional random process, where any combination of finite dimensions is a Gaussian distribution. Since Gaussian distribution is a distribution of random variables, Gaussian process can be completely determined by its mean function and covariance function. Therefore, Gaussian process has also been widely used in surrogate-assisted evolutionary algorithms.

Given a dataset containing *n* samples (*x*_*i*_, *y*_*i*_), *i* ∈ 1,2,…, *n*, the fitness *y* for any candidate solution *x* is regarded as *μ*+*ε*(*x*) in the GP model, where *ε*(*x*) obeys the distribution *N*(0, *σ*^2^).

(3) $$\begin{array}{c} \mu =k\left(x\right)K^{-1}y,\\ \sigma^{2}=\kappa \left(x\right)-k{\left(x\right)}^{\text{T}}K^{-1}k\left(x\right), \end{array}$$

*K* is the matrix, whose element is *K*_*ij*_=*C*(*x*_*i*_, *x*_*j*_). *k*(*x*)=[*C*(*x*, *x*_1_), *C*(*x*, *x*_2_),…,*C*(*x*, *x*_*n*_)]^T^. *X*=(*x*_1_, *x*_2_, ⋯, *x*_*n*_) is the input value of the sample point, and *κ*(*x*) is denoted as covariance, where *C*(·) is the covariance function. The covariance function can be written as follows:

(4) $$\begin{array}{c} C\left(x,x_{i}\right)=\sigma_{f}^{2}\left(1+\frac{\sqrt{3}r}{\sigma_{l}}\right)\text{exp}\left(-\frac{\sqrt{3}r}{\sigma_{l}}\right), \end{array}$$

where $r=\sqrt{\left(x-x_{i}\right){\left(x-x_{i}\right)}^{\text{T}}}$. *σ*_*f*_ and *σ*_*l*_ are denoted as hyperparameter. Therefore, the predicted values and variances of candidate solutions *x* can be derived as follows:

(5) $$\begin{array}{c} \hat{y}\left(x\right)=u\left(x\right)+k\left(x\right)K^{-1}\left(y-Iu\right), \end{array}$$

(6) $$\begin{array}{c} s^{2}\left(x\right)=\sigma^{2}\left(1+k^{T}\left(x\right)Kk\left(x\right)+\frac{{\left(1-I^{T}Kk\left(x\right)\right)}^{2}}{I^{T}K^{-1}I}\right), \end{array}$$

where I is the identity column vector.

In (5) and (6), $\hat{y}\left(x\right)$ and *s*^2^(*x*) are the mean prediction function and variance prediction function of Gaussian process regression, respectively. It can be seen that the prediction value of the variance prediction function at the point *x* is zero. The greater the distance from the existing sample point, the greater the prediction variance. Therefore, Gaussian process regression is an interpolation model about sample points. In addition, the distribution at prediction point *x* should meet the following requirements: *y*(*x*) ~ *N*(*u*(*x*), *s*^2^(*x*)) , so we can have

(7) $$\begin{array}{c} P\left(y\left(x\right)\leq t\right)=\Phi \left(\frac{t-u\left(x\right)}{s\left(x\right)}\right), \end{array}$$

where Φ(*x*) is the Gaussian cumulative density function. P(*y*(*x*) ≤ *t*) indicates the probability that *y*(*x*) is less than or equal to *t* at given *x*. Therefore, it can be given the confidence interval of *y*(*x*) with probability 1 − *α* at the point *x*.

(8) $$\begin{array}{c} y\left(x\right)\in \left[u\left(x\right)-\Phi^{-1}\left(1-\frac{\alpha }{2}\right)s\left(x\right),u\left(x\right)+\Phi^{-1}\left(1-\frac{\alpha }{2}\right)s\left(x\right)\right]. \end{array}$$

Although Gaussian process has been widely used in solving expensive multiobjective optimization problems, it has encountered some bottlenecks in solving high-dimensional expensive problems. In addition, the existing infilling criterion [33] cannot be well applied to high-dimensional problems, and the time of constructing Gaussian process model will also increase relatively with the increase of training samples. Compared with low-dimensional problems, constructing Gaussian process model on high-dimensional problems requires more training samples. Therefore, the construction of GP model on high-dimensional problems becomes more time-consuming.

### 3.2. Radial Basis Function

Radial basis function (RBF) [25,31] is a scalar function whose value only depends on the distance from the origin. It is generally defined as a monotonic function about the radial distance between the sample and the data center. RBF kernel is one of the commonly used kernel functions. In essence, the radial basis function model is formed by linear superposition. Its formation process is denoted as follows: first, input a sample set, then select the corresponding radial basis function, and finally calculate the weighted sum of the radial basis function values between unknown points and all sample points, to obtain the predicted response value of the test point.

Since the independent variable of radial basis function is the Euclidean distance between the testing set and the sample set, this can well transform the multidimensional problem into a one-dimensional problem with only the independent variable of Euclidean distance. Given *n* different samples *x*_1_, *x*_2_,…, *x*_*n*_ ∈ *R*^*D*^ and their corresponding function value *f*(*x*_1_), *f*(*x*_2_),…, *f*(*x*_*n*_) where *n* and *D* are arbitrary integers. Thus, we can have the following:

(9) $$\begin{array}{c} f\left(x\right)=\sum_{i=1}^{n}\lambda_{i}\varphi \left(\left|x-x_{i}\right|\right)+p\left(x\right)\;x\in R^{D}, \end{array}$$

where the coefficient *λ*_*i*_, *i*=1,2,…, *n* denotes the weight of the first *i* − th basis function; |·| is the Euclidean norm in *R*^*D*^; the degree of *p* is not greater than *m* from the polynomial space. It can be expressed as the linearity of the function *x*_1_^*k*_1_^, *x*_1_^*k*_2_^,…, *x*_1_^*k*_*D*_^, *x* ∈ *R*^*D*^, where there are many choices of kernel function *φ*(*x*) in radial basis function, such as linear, cubic, thin plate spline, and Gaussian. In our model, we adopted the Gaussian as kernel function, which can be written as follows:

(10) $$\begin{array}{c} \varphi \left(x\right)={\text{e}}^{-\delta x^{2}}, \end{array}$$

where *δ* > 0 and is a constant.

The unknowns (*λ*_1_, *λ*_2_,…, *λ*_*n*_) ∈ *R*^*D*^ in the radial basis function are obtained by solving the following:

(11) $$\begin{array}{c} \left(\begin{array}{cc} \varphi & p\\ p^{T} & 0 \end{array}\right)\left(\begin{array}{c} \lambda \\ C \end{array}\right)=\left(\begin{array}{c} F\\ 0 \end{array}\right). \end{array}$$

In a system of linear equations, *φ* represents a matrix of size *n* × *n*, where *φ*_*ij*_=*φ*(|*x*_*i*_ − *x*_*j*_|). In (11), **p**=(*x*_1_^*T*^*I*, *x*_2_^*T*^*I*,…*x*_*n*_^*T*^*I*)^*T*^, *λ*=(*λ*_1_, *λ*_2_,…,*λ*_*n*_)^*T*^, *C*=(*b*_1_, *b*_2_,…,*b*_*n*_)^*T*^, and F=(*f*(*x*_1_), *f*(*x*_2_),…,*f*(*x*_*n*_))^*T*^.

It can be seen that the RBF model established through the previously mentioned analysis can replace the expensive function to predict the individual fitness value in the process of algorithm optimization and performs best for different degrees of nonlinear problems with small-scale and noisy training datasets. Therefore, radial basis function is used to approximate the original problem in this paper and assist evolutionary algorithm optimization, to reduce the consumption of computing resources.

## 4. Integrated Surrogate Models for Noisy MOPs

As we all know, radial basis function model performs best for different degrees of nonlinear problems on small-scale and noisy training datasets but is insensitive to the increase of decision-space dimension, while Gaussian process regression model can provide prediction fitness and uncertainty evaluation. Therefore, an adaptive weighted strategy based integrated surrogate models is proposed to solve noisy multiobjective evolutionary problems in this paper.

### 4.1. Proposed Adaptive Weighted Strategy

The choice of surrogate-assisted model is very important to the good performance of surrogate-assisted evolutionary algorithm. RBF model and GPR model are the two most popular surrogate-assisted models. RBF model performs best for different degrees of nonlinear problems on small-scale training datasets and is insensitive to the increase of decision-space dimension [34]. GPR model can provide prediction fitness and uncertainty evaluation. Therefore, the combination of these two kinds of information can prevent the search from falling into local optimization. These characteristics are very attractive and promising for solving high-cost or expensive optimization problems. Although the integrated model has good convergence ability, it is difficult to solve the multimodal problem and noisy problem due to the lack of uncertainty evaluation [35].

Integrated surrogate models (ISM) are a kind of ensemble of surrogate models (EM). It is an integrated model composed of a series of surrogate-assisted models by weighting and combining. It can make use of the advantages of single surrogate-assisted model to effectively improve the robustness of prediction. The mathematical expression of surrogate models is described as follows:

(12) $$\begin{array}{c} \hat{y}=\sum_{i=1}^{N}w_{i}\left(x\right)y_{i}\left(x\right), \end{array}$$

where *w*_*i*_(*x*) is denoted as weight and obeys ∑_*i*=1_^*N*^*w*_*i*_=1. $\hat{y}$ and *y*_*i*_(*x*) represent the predicted values of EM and the integrated surrogate-assisted model, respectively.

The key step of constructing EM is to calculate the weight of each surrogate-assisted model. The prediction accuracy is positively proportional to the weight coefficient. According to the method of calculating weight, the existing weight strategies can be divided into two categories, including weighted average and point-by-point weight. The main principle of calculating the weight coefficient in the weighted average is to assign a weight to each subsurrogate-assisted model according to its global performance. Compared with the global error measurement, the weight of a single surrogate-assisted model for the point-by-point weight is calculated using the local error measurement, which means that the weight of each subsurrogate-assisted model will change in the whole sample space. Compared with the average weight method, the point-by-point weight method allows flexible adjustment of local weight coefficients in the sample space, which can better capture the local characteristics of the objective function, but it will increase the running time and is affected by noise.

Although the integrated model can obtain more accurate prediction values than a single model, and the effectiveness of the integrated model has been proved by theory, it is very difficult to fully meet the theoretical conditions of integrated surrogate model in practical applications, so it is difficult to ensure better generalization ability than a single model [36]. In our paper, an adaptive integration surrogate-assisted model is designed, which is shown in Table 1. First, calculate the number of samples in Step 1; calculate the Euclidean distance between the new sample point and all samples in the sample-set in Step 2; then, complete the division of training samples and test samples in Step 3, where 2/3 samples closest to the Euclidean distance of the new sample are selected as the training set to train each surrogate-assisted model. The remaining 1/3 samples are used as the test sample set to test the performance of the model. In Step 4, the training set divided in Step 3 is used to train RBF model and Gaussian process model, respectively. In Step 5, the performance of each surrogate-assisted model is tested on the test set, and the error of each surrogate-assisted model is calculated, respectively. In Step 6, the weight of each surrogate-assisted mode is updated. The weight of each surrogate-assisted model is equal to the ratio of the test error of the surrogate-assisted model in the test set to the sum of the test errors of all surrogate-assisted models. Update the corresponding weight according to the latest test error, where the sum of the weights of all surrogate-assisted models is 1. The method of updating the weight of the surrogate-assisted model by testing the error can adjust the weight at any time according to the performance of the surrogate-assisted model, so that the weight of the surrogate-assisted model with smaller error is greater, so as to enhance the prediction accuracy and prediction stability of the integrated surrogate-assisted model Finally, each surrogate-assisted model is used to predict and output the new predicted samples in Step 7, and then, the weighted sum of all the predicted output values is used as the output value of the final integrated model. It is worth noting that the training time of the integrated surrogate-assisted model can be reduced because not all samples are used in the training of the integrated model.

The next parent population need to be selected by some convergence selection strategy in the offspring generation, where we use crossover and mutation to produce the offspring solution.  Table 2 shows the main steps of offspring selection.

### 4.2. Indicator-Based Multiobjective Evolutionary Algorithm

The indicator-based multiobjective evolutionary algorithm adopts a fitness assignment scheme to rank the population members according to their usefulness regarding the optimization goal. The scheme is unique and simple and does not use the traditional diversity protection strategy, which makes the evolutionary algorithm have a good convergence and suitable for solving problems with a high dimension [36]. However, the algorithm performs poorly for some problems in maintaining the diversity preservation mechanism.

Performance indicator is a function that can use some preference information to assign a real number to any approximate solution set in many multiobjective evolutionary problems, so that the relative advantages and disadvantages of any two approximate solution can be judged according to the real number corresponding to each approximate solution set.

Since the binary performance index can assign a real function I(*x*_*i*_, *x*_*j*_) to any pair of approximate solution sets (*x*_*i*_, *x*_*j*_) in the object space, it can be directly used for fitness calculation, but there is a prerequisite that the used index must obey Pareto rule. Fitness allocation is to grade individuals in the population according to their utilization value in the process of seeking optimization objectives [37]. Therefore, the formula for calculating individual fitness using performance indicators in this paper is as follows:

(13) $$\begin{array}{c} F\left(x_{i}\right)=\sum_{x_{i}\in S/\left\{x_{i}\right\}}-e^{-I\left(\left\{x_{i}\right\},\left\{x_{j}\right\}\right)/c.k}, \end{array}$$

where *k* is a scaling factor greater than 0.5. The experimental results show that the algorithm can achieve better results when *k* = 0.05; *c* is the maximum of the absolute values of all indicators, namely, $c=\underset{x\in S}{\text{max}}\left|I\left(x_{i},x_{j}\right)\right|$.

The main steps of the indicator-based multiobjective evolutionary algorithm are expressed as follows.

1. First, initialize the population *Q*, take it as the initial population, and set an empty population *P*; set a variable *g* that holds the evolutionary generation.
2. The individuals in *P* and *Q* are merged into *R*, and the individuals in *R* are processed by the indicator-based fitness assignment scheme.
3. Execute the environment selection operation, and continuously repeat the following two steps: (a) select the individual with the smallest fitness value from *R* and delete it; (b) update the fitness of the remaining individuals; repeat the previously mentioned two steps until the number of remaining individuals in *R* is equal to the size of *P*, and then put the remaining individuals in *R* into the *P*.
4. Judge whether the variable *g* is greater than the maximum evolutionary generation or meets other termination conditions. If yes, stop evolution and output the noninferior solution in *P*, otherwise continue to execute.
5. Use tournament selection to select individuals from *P* and copy them into *Q*.
6. Perform cross-mutation operation on the individual in *Q* to generate offspring individuals and replace the parent individual in *Q* with a new generation of individuals; add 1 to the evolutionary generation (*g*=*g*+1) and turn to step (2).

### 4.3. U-Learning Sampling Approach

The larger the sample space, the higher the accuracy of the surrogate-assisted model. However, the construction of the surrogate-assisted model based on the larger sample space takes longer, so many scholars have successively studied the sampling size of the initial sample space of the surrogate-assisted model [37]. It is believed that, in general, the sampling size of the initial sample space should be 10 times the dimension of the variable of the multiobjective optimization problem.

For simulation-based optimization, we usually update the surrogate-assisted model step by step by iteratively selecting promising sampling points until the stop condition is met. A good sampling strategy should consider both global and local search. In recent years, more and more methods with novel ideas have been proposed [25,35]. In addition, some researchers focus on selecting multiple sampling points in each iteration. These new acquisition points can be simulated in parallel if parallel computing resources are available, and the number of iterations will be greatly reduced.

To select the training sample points of the model efficiently, it is usually necessary to adopt an adaptive method to select the appropriate learning function and convergence conditions. In this paper, U-learning (Table 3) function *U*(*x*)=|*u*(*x*)/*σ*(*x*)| is adopted as a sample point tool, which represents the probability that the positive and negative states of the sample output response *y*=*f*(*x*) are misclassified. The smaller the value of U-learning function, the greater the probability of sample points being misclassified. Therefore, the sample points with smaller value are selected as new training points $x_{\text{new}}=\underset{x\in S}{\text{argmin}}U\left(x\right)$, where *S* is the candidate sample pooling.

Literature [37] proves that the probability of sample points being correctly classified is 97.7% if *U*(*x*) is equal to 2, so it can be considered that the constructed Integrated surrogate-assisted model has more than 97.7% probability of correct prediction of sample points when *U*(*x*) > 2. Therefore, it is determined that the convergence condition of sample point is $\underset{x\in S}{\text{min}}U\left(x\right)\geq 2$.

## 5. Experiment and Simulation Analysis

### 5.1. Comparison Models

To verify the effectiveness of our proposed algorithm, we limit the number of real evaluations of the test problem to simulate the scenario of solving expensive MOPs. In addition, the existing popular surrogate-assisted evolutionary algorithms are also selected to evaluate its effectiveness. Next, we will briefly describe these selected comparison algorithms.

 
*parEGO* [38]: it is the first time to use EGO to solve multiobjective optimization problems parEGO. It divides the MOP problem into several subproblems evenly. In other words, the MOP problem is transformed into a single-objective optimization problem, randomly selects one subproblem at a time, and takes the expected improvement index (EI) as the sample selection strategy to select the solution for real evaluation. In addition, parEGO also limits the size of the training set to reduce modeling time.
 
*SMS-EGO* [39]: it uses the lower confidence bound to delimit the dominant relationship of the solution, and divide the obtained solution into non-*ε* dominant solution, *ε* dominant solution, and dominant solution. For non-*ε* dominant solution, SMS-EGO calculates its *S-metric* as the fitness. For *ε* dominant solution and dominant solution, SMS-EGO assigns a penalty value as fitness. The farther away from the nondominant solution, the greater the penalty. Based on this selection strategy, the solution for real evaluation is selected. Although SMS-EGO performs well, its running time is particularly long because it needs to calculate a lot of *S-metric*.
 
*MOEA/D-EGO* [40]: it clusters the solutions of the evolutionary algorithm and then uses EI as the selection strategy to select a solution in each cluster for real evaluation. In addition, it also uses the fuzzy clustering method to establish the surrogate-assisted model, which reduces the modeling time while using all the real evaluated solutions. Compared with parEGO, MOEA/D-EGO runs relatively faster because it selects solutions in batches each time, rather than just selecting a single solution.
 
*K- RVEA* [41]: the more contribution of this algorithm is in the model management strategy. It assigns the candidate solution obtained this time and the candidate solution obtained last time to a group of reference vectors, respectively. If the change in the number of inactive reference vectors is less than a certain threshold, uncertainty strategy is used as the basis for solution selection. Otherwise, the angle penalty distance is used as the basis for solution selection. In addition, when updating the model, K-RVEA will filter the solution to limit the size of the training set, to reduce the modeling time.
 
*CSEA* [42]: it uses the artificial neural network to predict the dominance relationship between candidate solutions and reference solutions. The uncertainty information in prediction is considered together with the dominance relationship to select promising solutions using the real objective functions.

### 5.2. Measurement Indicators

When the Pareto optimal solution set is obtained by the optimization algorithm, the advantages and disadvantages need to be analyzed by comparing the performance indexes. The performance index mainly evaluates the proximity between the nondominated solution set and the real optimal Pareto-front and the distribution and diversity of the solution set [43–45]. Therefore, some measurement indicators are proposed to evaluate the quality of the solutions obtained during optimization, to evaluate the quality of the algorithm. The performance evaluation indicators of the solution set of multiobjective optimization algorithm is mainly divided into convergence, uniformity, and spread. *Convergence* [46] reflects the difference between the solution set obtained by the optimization algorithm and the real Pareto-front. It is generally hoped that the solution set obtained is as close to the real Pareto front as possible. *Uniformity* reflects the degree of uniformity of the distribution of individuals in the solution set. Generally, it is hoped that the solution set obtained will be distributed as evenly as possible on Pareto front. *Spread* reflects the wide distribution degree of the whole solution set in the object space. Generally, it is hoped that the obtained solution set will be distributed on Pareto front as widely and completely as possible. Therefore, the performance evaluation indicators selected in this paper are shown as follows.

Generational distance (GD) is the average minimum distance from each point in the solution set *P* to the real solution set *P*^*∗*^. The smaller the generational distance value, the better the convergence.
  (14) $$\begin{array}{c} GD\left(P,P\right)=\frac{\sqrt{\sum_{y\in P}\text{min}x\in p^{*}\text{dis}{\left(x,y\right)}^{2}}}{\left|P\right|}, \end{array}$$
where *P* is the solution set obtained by the evolutionary optimization algorithm and *P*^*∗*^ is a set of uniformly distributed reference points sampled in the real Pareto front; dis(*x*, *y*) represents the Euclidean distance between the points y in the solution set *P* and the point *x* in the sample reference set *P*^*∗*^.
Inverted generational distance (IGD) [47]represents the mean value of the nearest individual from the reference point. The smaller the inverted generational distance value, the better the convergence performance.
  (15) $$\begin{array}{c} IGD\left(P,P^{*}\right)=\frac{\sum_{x\in P^{*}}\underset{y\in P}{\text{min}}\text{di}s\left(x,y\right)}{\left|P^{*}\right|}. \end{array}$$
Spacing is to measure the minimum distance standard deviation from each solution to other solutions. The smaller the spacing value, the more uniform the solution set.
  (16) $$\begin{array}{c} \text{Spacing}\left(P\right)=\sqrt{\frac{1}{\left|P\right|-1}\sum_{i=1}^{\left|P\right|}{\left(\bar{d}-d_{i}\right)}^{2}}, \end{array}$$
where $\bar{d}$ is denoted as the mean of all *d*_*i*_.
Diversity metric (DM) [1,3,37] is designed to measure the spread of the obtained solution set.
  (17) $$\begin{array}{c} \Delta =\frac{d_{f}+d_{l}+\sum_{i=1}^{N-1}\left|d_{i}-\bar{d}\right|}{d_{f}+d_{l}+\left(N-1\right)\bar{d}}, \end{array}$$
where d_*f*_ and *d*_*l*_ represent the Euclidean distance between the extreme solution and the boundary solution of the obtained nondominated solution-set.
Hyper volume (HV) [1,3] is a performance metric for indicating the quality of a nondominated approximation set, where the super volume of the area formed by the nondominated front obtained after the optimization and the previously reference points. The larger the HV value, the better the comprehensive performance of the evolutionary optimization algorithm.

### 5.3. Parameter Settings

For the sake of fairness, all comparison algorithms use the original default parameters. Since the comparison algorithms selected in this paper are based on surrogate-assisted evolutionary framework [12]. Therefore, the common parameter setting is consistent, for example, the number of initial population data is set to 100. In ZDT, the number of decision variables is set to 12, while that of DTLZ is set to 10. The number of object variables of ZDT and DTLZ are set to 2 and 3, respectively. The maximum real evaluation times of ZDT and DTLZ are set to 200 and 300, respectively. The setting probability of simulated binary crossover is 1.0 and its distribution index is 20. Polynomial mutation is selected as mutation operator, its probability is 1/d, and the distribution index is 20. The number of reference vectors is 300 for two targets and 595 for three targets. Each algorithm runs 30 times independently for each test problem.

The key parameters of our proposed adaptive integrated surrogate model are set as follows. Population size is 100, and its fitness scaling factor is set to 0.05. In this section, the number of individuals for sampling *η* is set to 5. The maximum number of generations is set to 30. It is worth noting that the improved weighting strategy in this paper can adjust the weight to realize the prediction based on noise data, where the variance of noise is set to 0.2.

In this paper, all experiments on the test function were run independently for 10 times, and the average value and standard deviation (STD) [31] of the results were collected and compared. All algorithms are implemented on MATLAB 2019a and run on Intel (R) core (TM) i7-3770 CPU @ 3.40 GHz and 8 GB of RAM on personal computers.

### 5.4. Qualitative and Quantitative Analysis

In the comparative experiment, the selected test function is DTLZ and ZDT [12,18,22]. They are designed for multiobjective optimization problems. One of its most important features is the extensible adaptability dimension, such as variable dimension and objective function dimension. All the problems in this set of test problems are continuous n-dimensional multiobjective optimization problems with box constraints, which are scalable in the fitness. In addition, the Pareto front of each problem is different.

#### 5.4.1. Comparison of Nondominated Solution for Different Algorithms

**Figure 3:**
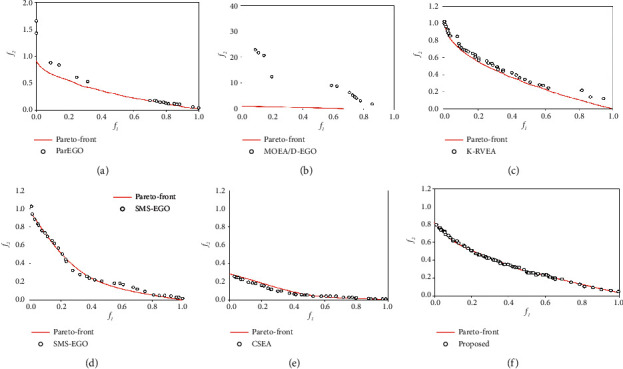
Comparison of nondominated solution for different algorithms on ZDT1. (a) ParEGO; (b) MOEA/D-EGO; (c) K-RVEA; (d) SMS-EGO; (e) CSEA; (f) our proposed.

**Figure 4:**
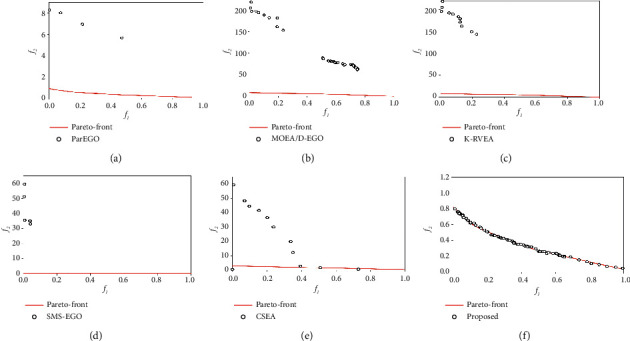
Comparison of nondominated solution for different algorithms on ZDT2 (*n* = 50). (a) ParEGO; (b) MOEA/D-EGO; (c) K-RVEA; (d) SMS-EGO; (e) CSEA; (f) our proposed.

ZDT1 and ZDT2 are relatively simple test problems. When the number of decisions is small (*n* = 10), they can achieve good convergence results. However, the performance of MOEA/D-EGO and K-RVEA decreased sharply with the increase of the number of variables. When *n* = 20, parEGO performs slightly better than MOEA/D-EGO and K-RVEA, as shown in Figure 3, but IGD exceeding 10 means that its convergence is still very poor.  Figure 4 shows the comparison of nondominated solution sets of different algorithms to obtain the optimal IGD value on ZDT1 (*n* = 50). When *n* = 50, parEGO, MOEA/D-EGO, and K-RVEA failed to find any solution on Pareto front. On the contrary, the performance of our proposed algorithm has been very stable from *n* = 10 to *n* = 50.

#### 5.4.2. Comparison of Measurement Indicators for Different Algorithms

Tables 4 and 5 respectively, show the inverted generational distance (IGD) and hyper volume (HV) of different comparison algorithms, where the number in brackets represents the standard deviation of the index, and bold indicates that the value is the best on this test problem. In addition, we use the average results of 30 independent runs for performance analysis. The symbols “+,” “−,” and “≈” indicate that our proposed weighted integrated surrogate model is statistically significantly superior to, inferior to, and almost equivalent to comparison models, respectively. The significance level of rank sum test is 0.05.

As shown in Table 4, only our proposed algorithm reached the near convergence state on DTLZ 1, while parEGO and MOEA/D-EGO performed poorly. On DTLZ2, K-RVEA performs best, and all algorithms can converge. On DTLZ3, all algorithms fail to converge, and our proposed algorithm is close to convergence. The performance of DTLZ4 is similar to that of DTLZ2. Our proposed algorithm is close to the convergence state, while other comparison algorithms have converged; on DTLZ5, all comparison algorithms can converge, where MOEA/D-EGO performed better. In addition, all comparison algorithms failed to converge on DTLZ6.

In Table 5, on the DTLZ1 problem, only our proposed algorithm performs best, but it fails to converge and it is relatively close. On DTLZ2, parEGO performs best and completes convergence, and our proposed algorithm is still close to convergence. DTLZ3 fails to reach the convergence state. The performance of DTLZ4 is similar to that of DTLZ2. On the DTLZ5 problem, the performance of all algorithms is similar, and only parEGO is slightly worse. All algorithms on DTLZ6 fail to converge.

Overall, our proposed algorithm in this paper performs better on the five-dimensional problem, especially on the more difficult DTLZ1 and DTLZ3 problems. Compared with the other two classical algorithms, our proposed algorithm still has an order of magnitude advantage in IGD performance. Our proposed algorithm is not satisfactory on the relatively simple DTLZ2 and DTLZ4 problems. In most cases, it is inferior to K-RVEA and CSEA. It is believed that our proposed algorithm has high convergence rate in population evolution, and has high IGD in most cases, which is consistent with the iterative curve.

**Figure 5:**
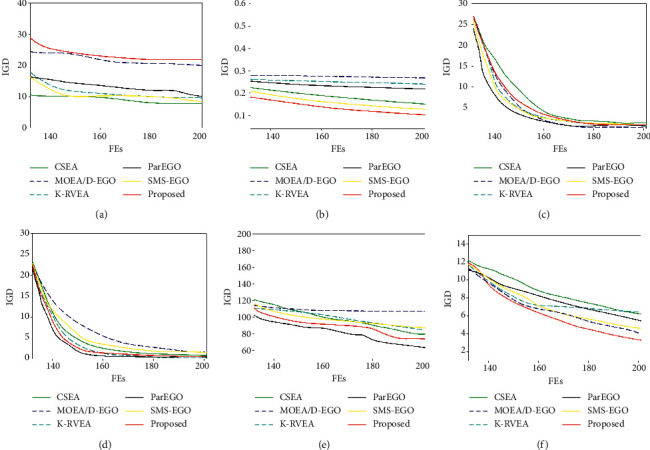
Comparison of convergence curve for different algorithms on ZDT problems. (a) ZDT1; (b) ZDT2; (c) ZDT3; (d) ZDT4; (e) ZDT5; (f) ZDT6.

**Figure 6:**
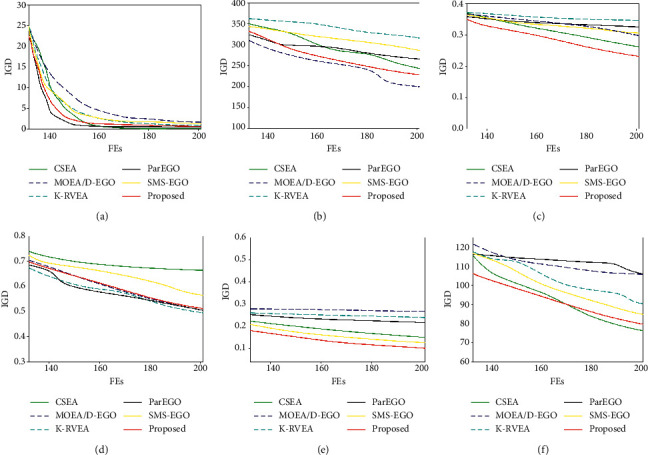
Comparison of convergence curve for different algorithms on DTLZ problems. (a) DTLZ 1; (b) DTLZ 2; (c) ZDT 3; (d) DTLZ 4; (e) DTLZ 6; (f) DTLZ 6.

Figures 5 and 6 show the convergence curves of our proposed algorithm and comparison algorithms on ZDT and DTLZ problems, where the abscissa is the real function evaluation times (Fes) and the ordinate is the IGD index. It is worth noting that, due to space constraints, we have only selected some problems for analysis. For ZDT test problem, the results of IGD and HV show that our proposed algorithm performs better when the experimental settings are consistent. The convergence curve shows that the convergence effect of our proposed algorithm is better than the comparison algorithm in most cases. Only on ZDT 1–3 problems, the convergence effect of our proposed algorithm is as small as parEGO before 170 real evaluations. Since the ZDT1–3 problems are relatively simple, our proposed algorithm is easy to find a better solution than the existing population in the initial stage. If the comparison algorithms select the solution according to the convergence, the convergence speed will be very fast. However, it is relatively easy to find a better solution than the existing population in the region close to the real PF, so the model needs to be able to better simulate the region near the real PF. Different from parEGO, our proposed algorithm selects solutions based on diversity, which is more inclined to increase the diversity of solution set, so that the surrogate-assisted model can better simulate the region near the optimal solution of the current population. Therefore, the convergence effect of the first 30th times of the convergence curve of our proposed algorithm is as small as that of parEGO, MOEA/D-EGO, and CSEA, and the later convergence effect is better than it.

For the DTLZ test problem, IGD, HV results and convergence curves show that our proposed algorithm performs better on most test problems when the experimental settings are consistent. For the DTLZ4 test problem, our proposed algorithm has the same effect as K-RVEA, but our proposed algorithm is not as good as MOEA/D-EGO for the DTLZ6 test problem.

#### 5.4.3. Ablation Analysis for Noisy Treatment

**Figure 7:**
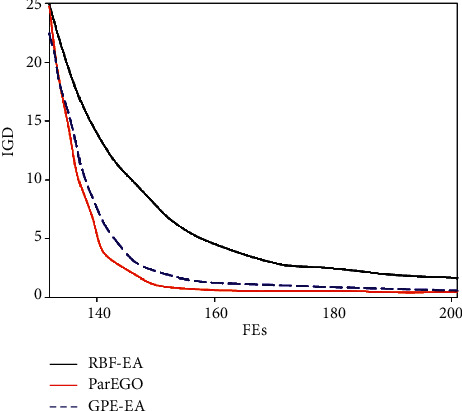
Comparative experimental curve for ablation analysis.

Based on the indicator-based multiobjective evolutionary framework, our proposed algorithm introduces the weighted combination of radial basis function and Gaussian process regression. In other words, two different surrogate-assisted models are linearly combined to improve the optimization performance. To analyze the performance of this optimization strategy, we used ablation analysis to explain this difference. The comparative experimental curve is shown in Figure 7, where RBF-EA, GPR-EA, and Both-EA denote a radial basis function, Gaussian process regression, and the weighted combination, respectively.

Since DTLZ belong to the test problems that are sensitive to noises, we only choose DTLZ to analyze the performance of our algorithm. The results are shown in Table 6. N-Both-EA is denoted as the version without noise treatment. It can be seen that the processing results of noisy data and clean data are quite different; that is to say, noise has a great impact on the performance of the evolutionary algorithm. However, our proposed algorithm has higher precision than other comparison algorithms, which is attributed to our proposed algorithm with the ability of noise removal. Therefore, it is very necessary to deal with the noises for multiobjective evolutionary algorithm.

The previously mentioned analysis shows that the designed model in this paper has higher performance than that using only a single strategy, which is helpful to improve the performance. In addition, through the comparison between our proposed algorithm and the other comparison algorithms, it is found that our proposed algorithm achieves better performance than the compared algorithms on noisy DTLZ and ZDT problems.

## 6. Conclusion

Radial basis function model performs best for different degrees of nonlinear problems on small-scale and noisy training datasets but is insensitive to the increase of decision-space dimension, while Gaussian process regression model can provide prediction fitness and uncertainty evaluation. Therefore, an adaptive weighted strategy based integrated surrogate models is proposed to solve noisy multiobjective evolutionary problems in this paper. Based on the indicator-based multiobjective evolutionary framework [7], our proposed algorithm introduces the weighted combination of radial basis function and Gaussian process regression, and U-learning sampling scheme is adopted to improve the performance of population in convergence and diversity and judge the improvement of convergence and diversity. Finally, the effectiveness of the proposed algorithm is verified by 12 benchmark test problems, which are applied to the hybrid optimization problem on the construction of samples and the determination of parameters. The experimental results show that our proposed method is feasible and effective [48–51].

## Figures and Tables

**Table 1:** Main steps of integrated surrogate-assisted model.

**Input**: sample-set *D*, the predicted sample *x*
**Output**: the model output value *y* of the sample *x* to be predicted;
**Step 1**: calculate the number of samples in *D*, which is recorded as *S*_*D*_;
**Step 2**: for *i*=1 to *S*_*D*_ do
Calculate the Euclidean distance *d*_*i*_ between the sample *x* and the *x*_*i*_;
End for
Select the nearest 2/3 samples closest to the Euclidean distance of *x* as the training set *D*_train_ and all the remaining samples as the test set *D*_test_;
**Step 3**: Gaussian process model *M*_1_ and RBF neural network model *M*_2_ are trained on the training set *D*_train_, respectively
**Step 4**: for *i*=1 to 2 *S*_*D*_ do
Calculate the test error *E*_*i*_ of the model *M*_*i*_ on the test set *D*_test_;
End for
**Step 5**: for *i*=1 to 2 do
Calculate the weight of surrogate-assisted model *M*_*i*_, and *w*_*i*_=*E*_*i*_/∑_*k*_*E*_*k*_;
End for
**Step 6**: outputs the final predicted value *y*=∑_*k*_*w*_*i*_*y*_*i*_, where *y*_*i*_ is the predicted value of the model *M*_*i*_ at *x*;

**Table 2:** Main steps of offspring selection.

**Input**: sample-set *D*, the predicted sample *x*
**Output**: offspring population *p*_0_;
**Step 1**: all individuals in sample-set *D* are evaluated by weight model;
**Step 2**: *N*/2: *N*/2 individuals are selected from *P*_*p*_ and *P*_*o*_;
**Step 3**: *N*/2 individuals are selected from all the remaining individuals in *P*_*p*_ and *P*_*o*_ by the *MSE* of all *m* surrogate for uncertainty selection;
**Step 4**: combine two groups of individuals as new *P*_*p*_ for updating.

**Table 3:** U-learning sampling strategy.

**Input**: sample-set *S*
**Output**: *x*_new_
**Step 1**: set *S*=*p*_*c*_
**Step 2**: while |*S*| > *η* do
Calculate *U*(*x*) and find the smallest sample individual *S* from the sample set;
Delete *S* from the set;
Calculate the fitness of the remaining individuals.
End while

**Table 4:** Comparison of inverted generational distance (IGD) for different algorithms.

Problems	ParEGO	MOEA/D-EGO	K-RVEA	SMS-EGO	CSEA	Proposed
ZDT1	4.7243*e* − 1 (2.08*e* − 1)−	1.7097*e* + 0 (3.74*e* + 0)−	8.2035*e* − 1 (1.15*e* − 1)−	3.7101*e* + 0 (3.14*e* + 0)−	8.2035*e* − 1 (2.14*e* − 1)−	6.2887*e* − 2 (2.73*e* − 2)
ZDT2	5.9465*e* − 1 (1.43*e* − 1)−	1.3118*e* + 0 (1.55*e* + 0)−	7.4677*e* − 1 (1.37*e* − 1)−	2.3611*e* + 0 (1.54*e* + 0)−	7.41257*e* − 1 (1.20*e* − 1)−	1.1052*e* − 1 (1.07*e* − 1)
ZDT3	536781*e* − 1 (1.61*e* − 1)−	1.4879*e* + 0 (1.73*e* + 0)−	8.1794*e* − 1 (1.59*e* − 1)−	3.4209*e* + 0 (1.63*e* + 0)−	8.17078*e* − 1 (1.60*e* − 1)−	1.9822*e* − 1 (1.88*e* − 1)
ZDT4	8.5326*e* + 1 (1.37*e* + 1)−	1.0688*e* + 2 (1.28*e* + 1)−	7.3940*e* + 1 (2.27*e* + 1)−	1.0711*e* + 2 (1.30*e* + 1)−	7.7943*e* + 1 (1.25*e* + 1)−	6.3493*e* + 1 (1.78*e* + 1)
ZDT5	6.4443*e* + 0 (8.96*e* − 1)−	6.2323*e* + 0 (1.99*e* + 0)−	5.4347*e* + 0 (2.27*e* + 0)−	5.2085*e* + 0 (1.87*e* + 0)−	5.8307*e* + 0 (2.17*e* + 0)−	3.2883*e* + 0 (8.31*e* − 1)
ZDT6	8.9775*e* + 1 (2.24*e* + 1)−	8.7589*e* + 1 (1.64*e* + 1)−	7.6495*e* + 1 (1.87*e* + 1)−	6.7327*e* + 1 (1.65*e* + 1)−	7.6085*e* + 1 (1.87*e* + 1)−	5.4726*e* + 1 (1.71*e* + 1)
DTLZ1	2.6802*e* − 1 (2.14*e* − 2)−	3.3203*e* − 1 (2.55*e* − 2)−	1.8420*e* − 1 (1.88*e* − 2)−	2.3251*e* − 1 (2.57*e* − 2)−	1.0320*e* − 1 (1.18*e* − 2)−	1.4977*e* − 1 (3.47*e* − 2)
DTLZ2	2.6622*e* + 2 (6.05*e* + 1)−	2.1185*e* + 2 (4.05*e* + 1)−	2.1205*e* + 2 (7.18*e* + 1)−	4.0287*e* + 2 (4.11*e* + 1)−	2.2505*e* + 2 (6.18*e* + 1)−	1.3896*c* + 2 (2.36*e* + 1)
DTLZ3	4.1291*e* − 1 (1.12*e* − 1)−	6.4259*e* − 1 (7.17*e* − 2)−	3.622*e* − 1 (9.93*e* − 2)≈	4.022*e* − 1 (7.07*e* − 2)−	3.6171*e* − 1 (9.93*e* − 2)≈	3.3670*e* − 1 (1.01*e* − 1)
DTLZ4	1.7326*e* − 1 (3.06*e* − 2)−	2.5429*e* − 1 (3.03*e* − 2)−	7.5002*e* − 2 (1.28*e* − 2)−	1.5019*e* − 1 (2.83*e* − 2)−	7.5662*e* − 2 (1.28*e* − 2)−	2.5984*e* − 2 (6.28*e* − 3)
DTLZ5	4.1258*e* + 0 (4.68*c* − 1)−	1.8576*e* + 0 (5.81*e* − 1)+	3.8069*c* + 0 (4.72*e* − 1)−	5.5814*e* + 0 (4.99*e* − 1)+	2.8071*c* + 0 (4.72*e* − 1)≈	2.7593*e* + 0 (4.34*e* − 1)
DTLZ6	3.3278*e* − 1 (6.28*e* − 2)−	2.3411*e* − 1 (9.23*e* − 2)−	1.0011*e* + 0 (1.10*e* − 1)−	3.0452*e* − 1 (8.93*e* − 2)−	1.8091*e* + 0 (1.10*e* − 2)≈	1.7815*e* − 1 (3.03*e* − 2)
+/−/≈	0/12/0	1/11/0	0/11/1	0/11/1	0/10/2	—

**Table 5:** Comparison of hyper-volume (HV) for different algorithms.

Problems	ParEGO	MOEA/D-EGO	K-RVEA	SMS-EGO	CSEA	Proposed
ZDTI	2.0311*e* − 1 (1.29*e* − 1)−	3.4343*e* − 1 (2.33*e* − 1)−	8.9466*e* − 2 (7.60*e* − 2)−	1.0688*e* − 2 (1.28*e* + 1)−	6.3940*e* − 1 (2.27*e* + 1)−	6.4567*e* − 1 (1.48*e* − 2)
ZDT2	4.0968*e* − 2 (3.81*e* − 2)−	9.4252*e* − 2 (1.02*e* − 1)−	4.9670*e* − 3 (1.05*e* − 2)−	3.2323*e* + 0 (1.99*e* + 0)≈	5.4347*e* + 0 (2.27*e* + 0)−	3.2907*e* − 1 (8.19*e* − 2)
ZDT3	1.9277*e* − 1 (1.16*e* − 1)−	2.4279*e* − 1 (2.35*e* − 1)−	1.0257*e* − 1 (1.19*e* − 1)−	8.7589*e* − 1 (1.64*e* + 1)−	5.1495*e* + 1 (1.87*e* + 1)−	5.2323*e* − 1 (1.39*e* − 1)
DTLZ2	1.8637*e* − 1 (3.65*e* − 2)−	1.3774*e* − 1 (4.64*e* − 2)−	3.5085*e* − 1 (2.65*e* − 2)−	4.3203*e* − 1 (2.55*e* − 2)≈	4.8420*e* − 1 (1.88*e* − 2)≈	4.3094*e* − 1 (4.84*e* − 2)
DTLZ4	2.1018*e* − 1 (6.82*e* − 2)≈	8.5741*e* − 3 (1.49*e* − 2)−	1.2883*e* − 1 (1.17*e* − 1)−	7.1185*e* + 2 (4.05*e* + 1)−	2.1205*e* − 1 (1.18*e* − 1)≈	2.0978*e* − 1 (1.07*e* − 1)
DTLZ5	5.9209*e* − 2 (2.35*e* − 2)−	2.5012*e* − 2 (2.17*e* − 2)−	1.4107*e* − 1 (1.17*e* − 2)−	6.4259*e* − 1 (7.17*e* − 2)−	1.62 2*e* − 1 (6.93*e* − 3)≈	1.7894*e* − 1 (6.78*e* − 3)
DTLZ7	1.5591*e* − 1 (2.40*e* − 2)−	2.1222*e* − 1 (1.71*e* − 2)−	1.5166*e* − 1 (1.28*e* − 2)−	3.5429*e* − 1 (3.03*e* − 2)−	3.5002*e* − 1 (1.28*e* + 2)−	2.2066*e* − 1 (1.15*e* − 2)
+/−/≈	0/6/1	0/7/0	0/7/0	0/5/2	0/4/3	—

**Table 6:** Results of noisy treatment on the DTLZ problems.

Problems	RBF-EA	GPR-EA	N-Both-EA	Both-EA
DTLZ-2	1.373*e* − 1 (2.55e − 2)≈	4.8420*e* − 1 (1.88*e* − 2)≈	4.3094*e* − 1 (4.84*e* − 2)−	1.3094*e* − 1 (1.84*e* − 2)
DTLZ-3	4.1185*e* + 2 (4.05e − 2)−	2.1274*e* − 1 (1.18*e* − 1)≈	2.0914*e* − 1 (1.07*e* − 2)−	1.5978*e* − 1 (9.07*e* − 1)
DTLZ-4	6.7259*e* − 1 (7.17e − 2)−	1.67 2*e* − 1 (6.93*e* − 3)≈	1.8694*e* − 1 (6.17*e* − 3)≈	1.7490*e* − 1 (5.78*e* − 3)
DTLZ-5	3.5729*e* − 1 (3.03e − 2)−	3.7002*e* − 1 (1.28*e* + 2)−	2.2066*e* − 1 (1.15*e* − 2)≈	2.210*e* − 1 (1.75*e* − 2)
DTLZ-6	3.5429*e* − 1 (3.03e − 2)−	3.5002*e* − 1 (1.28*e* + 2)−	2.2066*e* − 1 (1.15*e* − 2)−	1.2021*e* − 1 (1.15*e* − 2)

## Data Availability

The labeled datasets used to support the findings of this study are available from the corresponding author upon request.
